# Supplementary material for: Content-rich biological network constructed by mining PubMed abstracts
Source: BMC Bioinformatics. 2004 Oct 8;5:147. doi: 10.1186/1471-2105-5-147 (PMC528731; doi:10.1186/1471-2105-5-147)
Supplement: Additional File 5 — The original Chilibot query results of the term "long-term potentiation (LTP)" and 22 other terms, limiting the latest references analyzed to the years 1990, 1995, 2000, and 2004. [file 1471-2105-5-147-S5.bz2 › chilibotAdditionalFile5/ltp2004/html/PKA_AMPA.html]

 


 **PKA** and **AMPA** 
  
Found 62 abstracts in PubMed,  **30 abstracts were retrieved and analyzed**.  


---

 Search Google  |
 PDF files only 
|  EDU domain only 

---

**Interactive relationship** (e.g. stimulation, inhibition, etc)

- These findings indicate that  **PKA**  phosphorylation is important in determining  **AMPA**  receptor surface expression and suggest a mechanism by which DA releasing drugs of abuse may directly tap into fundamental mechanisms that enable synaptic plasticity.  Ref: 14684450 Ann N Y Acad Sci, 2003
- Recent studies have shown that phosphorylation of  **AMPA**  receptors by cAMP dependent protein kinase  **PKA**  and calcium and calmodulin dependent protein kinase II CaMKII potentiates their activity, but phosphorylation of the receptor subunits may also affect their interaction with intracellular proteins, and their expression at the plasma membrane.  Ref: 11059799 Neurochem Res, 2000
- We found that  **PKA**  phosphorylation of the  **AMPA**  receptor subunits GluR4 and GluR1 directly controlled the synaptic incorporation of  **AMPA**  receptors in organotypic slices from rat hippocampus.  Ref: 12536214 Nat Neurosci, 2003
- Thus, endogenous synaptic  **AMPA**  currents in XII motoneurons are influenced by phosphorylation, specifically by  **PKA** , and dephosphorylation.  Ref: 12598595 J Neurosci, 2003
- The neuronal A kinase anchoring protein AKAP79 150 interacts with  **PKA**  and the calcium dependent protein phosphatase PP2B and is linked to the  **AMPA**  receptor GluR1 subunit by synapse associated protein 97 SAP97, a membrane associated guanylate kinase family protein.  Ref: 11943807 J Neurosci, 2002
- Hippocampal long term depression LTD is a calcium dependent downregulation of synaptic  **AMPA**  receptor currents associated with dephosphorylation of Ser845, a cAMP dependent protein kinase  **PKA**  site on GluR1.  Ref: 11943807 J Neurosci, 2002
- At the postsynaptic membrane of glutamatergic synapses, the cAMP dependent protein kinase  **PKA** , protein kinase C PKC, and calcineurin CaN anchoring protein AKAP79 150 is recruited to NMDA and  **AMPA**  glutamate receptors by postsynaptic density PSD 95 family membrane associated guanylate kinase MAGUK scaffold proteins.  Ref: 12177200 J Neurosci, 2002
- The serotonergic modulation of  **AMPA**  receptor currents was blocked by protein kinase A activators and occluded by  **PKA**  inhibitors.  Ref: 12149253 J Biol Chem, 2002

**Parallel relationship** (e.g. studied together, co-existance, homology, etc.)

- Regulation of DARPP 32 dephosphorylation at  **PKA**  and Cdk5 sites by NMDA and  **AMPA**  receptors distinct roles of calcineurin and protein phosphatase 2A.  Ref: 12065642 J Neurochem, 2002
- High frequency stimulation increased the fraction of dynamic terminals for 1 2 days, a process mediated by activation of  **AMPA**  receptors, protein kinase A  **PKA**  and protein synthesis.  Ref: 12692557 Nat Neurosci, 2003
- Intracellular perfusion with the catalytic subunit of  **PKA**  cPKA into preBötC inspiratory neurons progressively enhanced inspiratory drive currents and, in the presence of TTX, increased the inward currents induced by local ejection of  **AMPA** .  Ref: 12562968 J Physiol, 2003
- Thus,  **PKA**  phosphorylation of  **AMPA**  receptor subunits contributes to diverse mechanisms underlying synaptic plasticity.  Ref: 12536214 Nat Neurosci, 2003
- In rhythmically active brainstem slices from neonatal rats, we investigated whether synaptic  **AMPA**  receptor function could be modulated by changes in intracellular  **PKA**  activity, affecting inspiratory drive in hypoglossal XII motoneurons.  Ref: 12598595 J Neurosci, 2003
- Intracellular perfusion of the catalytic subunit of  **PKA**  potentiated endogenous synaptic and exogenously applied  **AMPA**  induced currents in XII motoneurons.  Ref: 12598595 J Neurosci, 2003
- 0 microg, the  **AMPA**  receptor blocker, cyanonitroquinoxaline dione CNQX, 1.25 microg, the generic glutamate metabotropic receptor antagonist, alpha methyl 4 carboxyphenyl glycine MCPG, the inhibitor of cAMP dependent protein kinase  **PKA** , Rp cAMPs 0.1 or 0.5 microg, or the inhibitor of the mitogen activated protein kinase MAPK, PD098059 10 or 50 microM.  Ref: 11099775 Behav Brain Res, 2000
- The present project was designed to investigate the role of protein kinase A  **PKA**  and protein kinase C PKC in the regulation of phosphorylation of the GluR1 subunits of  **AMPA**  receptors in the spinal cord of rats after capsaicin injection.  Ref: 14559367 Brain Res Mol Brain Res, 2003
- These results suggest that phosphorylation of postsynaptic  **AMPA**  receptors through the cAMP  **PKA**  pathway modulates both tonic and phasic excitatory amino acid synaptic transmission and excitability of inspiratory neurons in the preBötC and, therefore, regulates respiratory rhythm.  Ref: 12562968 J Physiol, 2003
- The results indicate that the effect of novelty on retrieval can not be observed if the major biochemical mechanisms of retrieval  **AMPA**  receptors,  **PKA** , MAPK are blocked, i.e. if the hippocampus was temporarily inactivated by drugs that inhibit those mechanisms.  Ref: 11099775 Behav Brain Res, 2000
- Our results suggest that increased phosphorylation of the GluR1 subunit of  **AMPA**  receptors contributes to central sensitization following acute peripheral inflammation, and the effect may occur at different phosphorylation sites through the activation of the  **PKA**  or PKC protein kinase cascades.  Ref: 14559367 Brain Res Mol Brain Res, 2003
- **PKA**  phosphorylation of  **AMPA**  receptor subunits controls synaptic trafficking underlying plasticity.  Ref: 12536214 Nat Neurosci, 2003
- Because  **AMPA**  receptor  **PKA**  phosphorylation and synaptic localization are regulated by similar NMDA receptor CaN signaling pathways linked to hippocampal long term depression, this regulation of AKAP79 150 postsynaptic targeting might be important for synaptic plasticity.  Ref: 12177200 J Neurosci, 2002
